# Evaluating the Importance of the Carotid Chemoreceptors in Controlling Breathing during Exercise in Man

**DOI:** 10.1155/2013/893506

**Published:** 2013-10-23

**Authors:** M. J. Parkes

**Affiliations:** School of Sport, Exercise & Rehabilitation Sciences, University of Birmingham, Edgbaston, Birmingham B15 2TT, UK

## Abstract

Only the carotid chemoreceptors stimulate breathing during hypoxia in Man. They are also ideally located to warn if the brain's oxygen supply falls, or if hypercapnia occurs. Since their discovery ~80 years ago stimulation, ablation, and recording experiments still leave 3 substantial difficulties in establishing how important the carotid chemoreceptors are in controlling breathing during exercise in Man: (i) they are in the wrong location to measure metabolic rate (but are ideally located to measure any mismatch), (ii) they receive no known signal during exercise linking them with metabolic rate and no overt mismatch signals occur and (iii) their denervation in Man fails to prevent breathing matching metabolic rate in exercise. New research is needed to enable recording from carotid chemoreceptors in Man to establish whether there is any factor that rises with metabolic rate and greatly increases carotid chemoreceptor activity during exercise. Available evidence so far in Man indicates that carotid chemoreceptors are either one of two mechanisms that explain breathing matching metabolic rate or have no importance. We still lack key experimental evidence to distinguish between these two possibilities.

## 1. Introduction 

Following earlier studies of the anatomy, sensory innervation, and cardiovascular functions of the carotid bifurcation region by De Castro [1], the respiratory functions of the carotid chemoreceptors were discovered in 1927 by accident [2] and won Corneille Heymans the 1938 Nobel Prize in medicine. The carotid chemoreceptors remain the only chemoreceptors known in Man to be stimulated by hypoxia and that in turn stimulate breathing. It has always been believed therefore that they must be key to explaining how breathing matches metabolic rate so well. Yet since 1927, the experimental evidence has failed to reveal whether they have any importance in Man.

**Figure 1:**
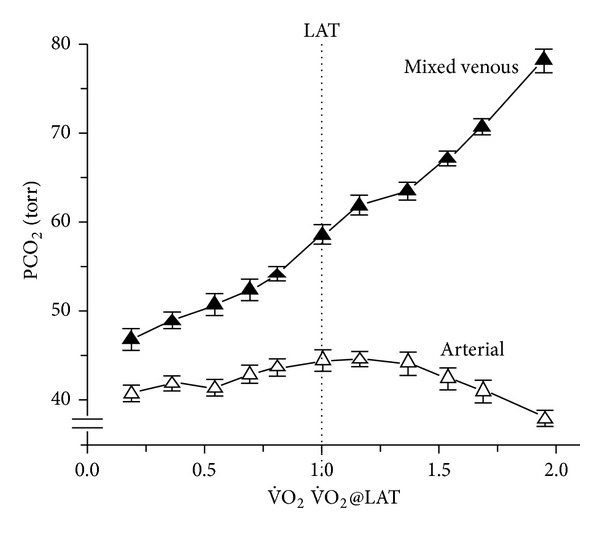
PaCO_2_ fails to rise above the normal range during exercise (unlike PvCO_2_). Mean ± se PaCO_2_ (brachial artery) and mixed venous PvCO_2_ (pulmonary artery), during incremental exercise to maximum, normalized to their mean lactic acidosis threshold (LAT) of 2.0 ± 0.0  LO_2_ min^−1^. 0.7 kW in 5 healthy subjects. Reproduced with permission from Sun et al. [75].

**Figure 2:**
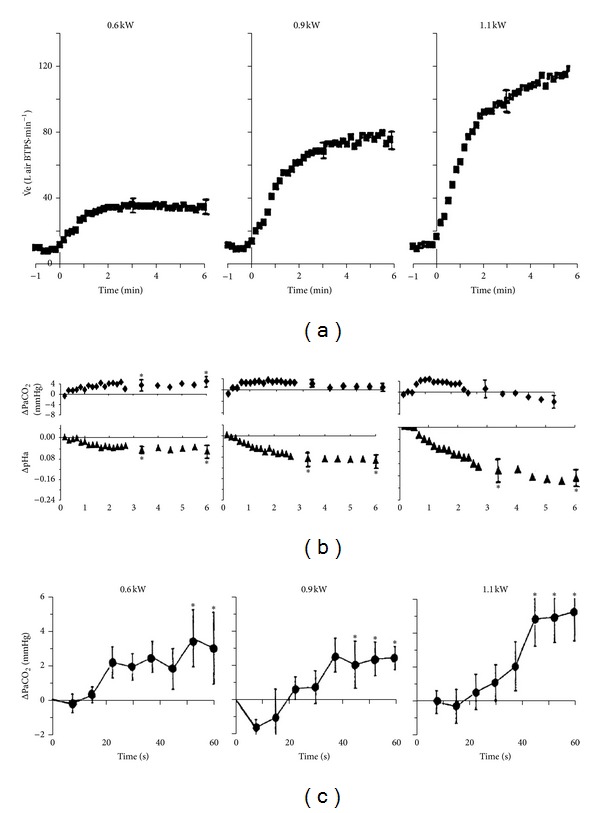
No detectable PaCO_2_ rise above the normal range during exercise even when frequently sampled. (a) Mean breathing. (b) Mean ± se change in brachial PaCO_2_ from resting values (ΔPaCO_2_) and mean change in pHa (ΔpHa) during rest followed by the 6 min exercise period (average values for the 8 subjects displayed in 10 s time bins) with cycling at ~0.6 kW (80% of their lactic acidosis threshold), ~0.9 kW, and ~1.1 kW (mean $\dot{\text{V}}{\text{O}}_{2}$ max. was 3.6 L O_2 _min^−1^ = 1.2 kW) in 8 subjects. (c) Mean ± se ΔPaCO_2_ during the first minute only. *P < 0.05  versus rest (repeated measures ANOVA). Reproduced with permission from Stringer et al. [68].

Between rest and maximum exercise in Man, metabolic rate (the rate of oxygen consumption, $\dot{\text{V}}{\text{O}}_{2}$) increases by up to ~23-fold, and breathing increases by up to ~35-fold to match (a hyperpnea) or exceed (a hyperventilation) metabolic rate. If breathing matches metabolic rate, the systemic arterial blood gases (the partial pressures of oxygen [PaO_2_] and of carbon dioxide [PaCO_2_]) should remain constant and the stability of PaCO_2_ is the simplest indication of how well matching occurs. Since they do match during exercise (Figures 1 and 2), there is no arterial blood gas signal to tell the carotid chemoreceptors what metabolic rate is. This has always been a fundamental problem in exercise physiology.

Julius Comroe wrote in 1944

* the history of respiratory physiology for the last 60 years has been a succession of attempts to explain all deviations from normal respiration by one theory … but no one theory yet offered can account for all or even a large part of the hyperpnea of muscular exercise, and each of these theories has failed to gain general acceptance because of its inability to explain this the most common and most powerful of all respiratory adjustments* [3]. **

Despite many attempts to address this problem and to evaluate the numerous other possible mechanisms involved (e.g., [4–10]), and despite the outstanding work on Man of Wasserman, Whipp et al. [11–14], the remarkable situation exists in 2012 where two reviews in the same journal still draw very different conclusions. Thus,

*the plasticity of the sensory response of the carotid body … are critical for ventilatory adaptations … as well as during exercise *[15] **

but

*stimuli postulated to act at … carotid … chemoreceptors are not primary mediators of the hyperpnea [16].*

This review identifies the key experimental evidence available in Man and what is still required to establish how important the carotid chemoreceptors are in controlling breathing in exercise in Man, the species in which we have most interest. It deliberately follows Bronowski's [17] dictum

* Students … are not here to worship what is known but to question it.*

## 2. Terminology

 Here the terms “breathing,” “ventilation,” and “minute ventilation ($\dot{\text{V}}\text{e}$)” will be used interchangeably and measured as minute ventilation ($\dot{\text{V}}\text{e}$, in litres air BTPS·min^−1^). Measurements of metabolic rate ($\dot{\text{V}}{\text{O}}_{2}$) are made at the mouth by indirect calorimetry in kilowatts (where kW = LO_2_ STPD·min^−1^ x an assumed fuel conversion factor of 0.3366), and rate of CO_2_ production ($\dot{\text{V}}\text{C}{\text{O}}_{2}$) is measured at the mouth as LCO_2_ STPD·min^−1^. External work rate is indicated as Watts (W_ew_). Measurements are cited as means ± SEM, and the number of subjects is indicated by  *n* = .

## 3. Carotid Chemoreceptors as Metabolic Rate or Mismatch Sensors and Their Ideal Location 

**Figure 3:**
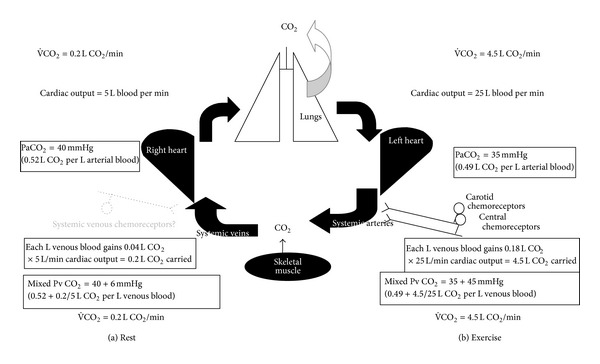
Systemic arterial chemoreceptors are ideally located anatomically to measure any mismatch but are in the wrong place to measure metabolic rate or metabolically produced CO_2_. Diagram of typical values of cardiac output, systemic arterial PaCO_2,_ and mixed PvCO_2,_ and of blood CO_2_ content at rest (a) and maximum exercise (b), with data from multiple sources. For clarity, the corresponding numbers for metabolic rates are omitted. Comparison of (a) and (b) shows that systemic arterial chemoreceptors are in the wrong place to measure metabolically produced CO_2_, because metabolically produced CO_2_ is carried via the systemic veins from muscle to air without PaCO_2_ rising.

The simplest system to explain how breathing matches metabolic rate so well requires something acting as a metabolic rate sensor. This sensor does not have to be a chemoreceptor. If it is, ideally it would somehow measure how much O_2_ has been extracted from arterial blood (the systemic arteriovenous difference in O_2_ content), and metabolic rate could be derived by combining this with knowledge of cardiac output (via the Fick principle). The fundamental difficulty, however, is that the known peripheral chemoreceptors are located only on the systemic arterial side. They are therefore in the wrong location to measure metabolic rate, because O_2_ has not yet been extracted and because CO_2_ has already been removed. Figures 1 and 3 show that it is the systemic venous, not arterial, blood gases that change proportionately with metabolic rate. Yet no cardiac, pulmonary, nor mixed venous chemoreceptors have yet been found in Man [11, 18].

Although in the wrong place to measure metabolic rate itself, carotid chemoreceptors are ideally located to indicate if any breath fails to match metabolic rate and hence to provide the necessary signal to fine-tune each breath with metabolic rate. A single mismatching breath would cause arterial blood gases to deviate from normal and provide a blood gas error signal to modify the next breath. The bigger the mismatch, the bigger the error signal. Although this error signal is inherently ambiguous (it cannot distinguish a mismatch caused by an inappropriate breath from a change in metabolic rate or from disproportionate changes in both), the system appears to be wired on the assumption that any mismatch is caused by hypoventilation rather than raised metabolic rate. This is because arterial hypoxia or hypercapnia stimulates breathing rather than reduce metabolic rate. The carotid chemoreceptors undoubtedly have the capability to signal any mismatch and have a response time fast enough to fine-tune each breath. All this is of clinical relevance [19]. But as shown below there is no evidence yet that they do perform this role, nor that this role makes any important contribution to the control of breathing in exercise.

## 4. What Is Necessary to Establish If Carotid Chemoreceptors Are the Metabolic Rate Sensor? 

Classical scientific method in neurophysiology (originating with Bernard [20] and Mill [21], and see also [22–26]) proposes that 3 conditions must be satisfied to establish whether any one sensor alone is the key metabolic rate sensor:

1. stimulation at rest of this sensor should mimic the maximum increase in breathing (100–150 L air·min^−1^) at maximum metabolic rate (~5 LO_2_·min^−1^),
2. ablation of this sensor should prevent breathing matching metabolic rate,
3. recorded activity of this sensor must increase and decrease proportionately with metabolic rate.

To establish the carotid chemoreceptors as the metabolic rate sensor, all three conditions must be satisfied. Either alone is inadequate; for example, (1) alone might also be achieved by an artificial stimulus, for example, a drug, (2) alone might merely represent some traumatic or accidental side effect, and (3) alone establishes only correlation but not causation. This review will consider each condition for carotid chemoreceptors, but before doing so, some further points must first be considered.

## 5. The Redundancy, Summation, and Multiplication Beliefs 

All ablation experiments in exercise so far fail to abolish breathing matching metabolic rate. Rather than concluding that the ablated factors are irrelevant, it is usually concluded instead that at least two sensor mechanisms must exist, each of which could mediate matching on its own, but at any moment one or more must contribute (i.e., both duplicate or are redundant). This is one of two common beliefs:

- the redundancy (or duplication) belief

*nature would not have designed such an important control system to rely on only one mechanism … so at least two factors must control …,*

(ii) the multiplication belief

*… hyperpnea of exercise … due to multiplication of a great number of discrete … impulses which are individually small but collectively large. *

These beliefs are automatically applied when interpreting carotid chemoreceptor experiments and are never challenged. Both are scientifically testable in Man, but there is no substantial experimental evidence yet for either.

The redundancy belief means that we must already accept that at least two mechanisms sense metabolic rate (i.e., one in addition to carotid chemoreceptors). This in turn comes up against Occam's razor

*(essentia non sunt multiplicanda praeter necessitatem):*

it is naïve to propose multiple mechanisms before first establishing one. Yet so far we cannot even establish one. No stimulation of a single mechanism has yet increased breathing by more than ~15 L·min-1 [27, 28], that is, to nowhere near that of maximum exercise. The principal interest in the redundancy belief, however, is the difficulty that it creates in interpreting the negative effect of an ablation experiment in Man, as further explained in Section 6.

The multiplication belief predicts that the more multiplication the interaction requires, the more vulnerable breathing must be to ablation of any single mechanism. Neither ablation of carotid chemoreceptors nor of any other mechanism has yet detected any such vulnerability of breathing in Man, see for example [29, 30].

## 6. Can Stimulation, Ablation, and Recording Establish That Carotid Chemoreceptors Are Not the Key Sensor in Man?

Falsifying any one of these three conditions could establish that carotid chemoreceptors are not the key sensor. Falsification, however, is difficult for two reasons. First, it is more difficult to deal with negatives (no change in response). For example, it is not yet possible to record neural activity directly from the carotid chemoreceptors in Man and hence Karl Sagan's

*“absence of evidence is not evidence of absence.” *

Ultimately, negatives are validated only by discovering what mechanism does satisfy all three conditions. Secondly, the safer conclusion from a negative result is initially “inconclusive without further experiments,” because either some experimental error occurred (e.g., the ablation was technically unsuccessful or some measurement error occurred), or because the ablated mechanism is either irrelevant or redundant.

Resolving “inconclusive” requires independent verification that ablation was successfully achieved, that no experimental errors occurred and then identification and ablation of the second (or more) mechanism(s). There are equal difficulties in identifying what might be the second duplicating mechanism (and the quest to identify the second mechanism is beyond the scope of this review). Only if ablating the second mechanism alone prevents matching, could this finally establish the irrelevance of the first mechanism and the importance of the second. Whereas if matching was prevented only by ablating both, both are important and duplicate. This review will also demonstrate that we are equally unable to establish definitively that carotid chemoreceptors are not the key metabolic rate sensor in Man.

## 7. The Ideal Experimental Design with Carotid Chemoreceptor Experiments

It is assumed that the same control system explains matching from rest to maximum exercise, rather than exercise being some special case. This is important for two reasons. First, whatever explains matching should be greater, hence easier to detect, as metabolic rate increases. Secondly, whatever explains matching must explain it over the large increments (~35-, ~23-, and ~21-fold) in the key variables ($\dot{\text{V}}\text{e}$, $\dot{\text{V}}{\text{O}}_{2}$, and $\dot{\text{V}}\text{C}{\text{O}}_{2}$) up to maximum exercise (≥1.9 kW). 

There is also the scientific tenet that all experimental results should be confirmed by independent laboratories (every “fact” should be referenced at least twice). Many of the crucial carotid chemoreceptor experiments cited here have only ever been attempted once. Furthermore, there is no agreement on the ideal number of subjects per study. Few use more than 10 (often for good practical reasons) and many do not even use 5. Statistical analysis can help (but not all apply it), although statistical power calculations are limited because they are for design rather than analysis, and because they too depend on the number of subjects. 

Positive results that are statistically significant (because the observed effect size is so large and its variance is so small), even with only two subjects, are important and have sufficient power. But even these have two problems. First, there cannot be complete confidence in variance being estimated from only 2 subjects. Secondly, there can be difficulty in convincing the scientific community that these 2 subjects are sufficiently representative of the population.

The particular problem with many of the key carotid chemoreceptor experiments on Man reviewed here, however, is that negative results occurred, few subjects were used, the observed effect size is small and the variance is large. For example, with only 2 subjects, the observed effect size is often not statistically significant. The difficulty then is in distinguishing a lack of power (false negative) from a true negative. Strictly, statistical power analysis indicates only that the design requires more subjects; which only confirms our original concern about low subject numbers.

## 8. Recording Experiments from Human Arterial Blood 

The principal physiological stimulants of the carotid bodies are hypoxia, hypercapnia, and acidity. Without the ability to record from carotid chemoreceptor activity in Man, measuring PaCO_2_, PaO_2_, and pHa levels is the only means of investigating the recording condition for carotid chemoreceptors; that is, are carotid chemoreceptors stimulated by raising metabolic rate? 

A fundamental requirement of a classical control system using feedback from a metabolic rate sensor to drive breathing is that arterial blood gases must change appropriately and proportionately with metabolic rate. To produce the “error” signal that drives breathing, they must change in the appropriate direction—PaO_2_ falling and PaCO_2_ rising, they should change in proportion to exercise intensity, and they *must remain changed* as long as metabolic rate is increased in order to sustain breathing at this new metabolic rate. 

**Figure 4:**
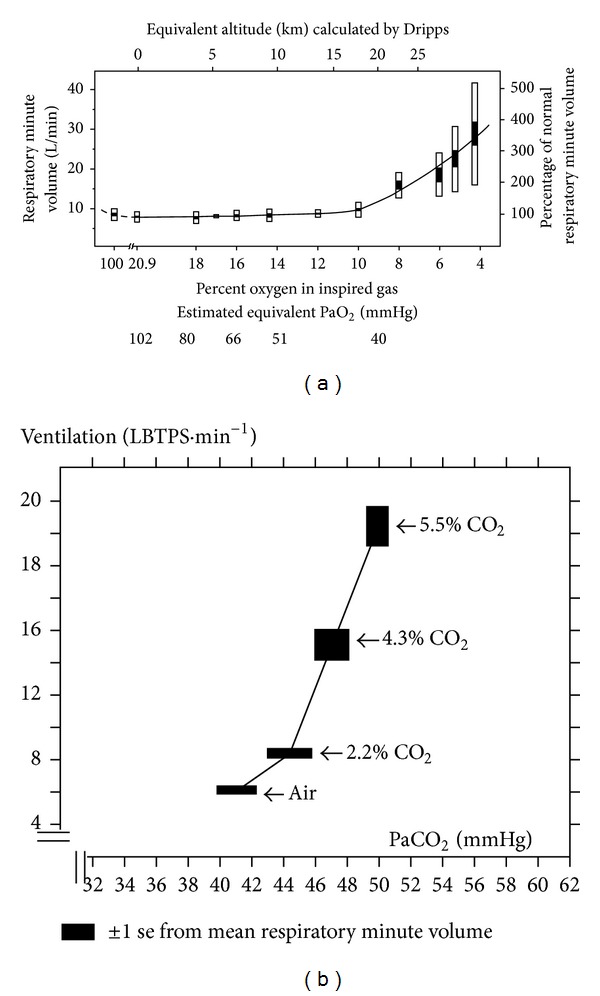
(a) Relative insensitivity of breathing at rest to artificially lowering PO_2_ in Man. Minute ventilation (± se solid bars, ± sd open bars) in normal subjects [87] as inspired oxygen is artificially lowered (strictly hypocapnic hypoxia exists once hyperventilation occurs). Equivalent PaO_2_ points are aligned on the FiO_2_ scale, with PaO_2_ estimated before hyperventilation occurs using the alveolar gas equation (assuming 760 mmHg barometric pressure, RQ = 0.8, PaO_2_ = PAO_2_, and PaCO_2_ = PACO_2_), and the point afterwards is estimated based on dynamic forcing experiments in isocapnia (courtesy of Dr. G. A. Balanos). Reproduced with permission from Dripps et al., [87]. (b) Sensitivity of breathing at rest to artificially raising PaCO_2_ in Man. Minute ventilation and PaCO_2_ (femoral) in 8 healthy men [88] while inhaling 0–6% CO_2_ in air at atmospheric pressure (mean slope is 2.5 L·min^−1^ mmHg^−1^ artificial PaCO_2_ rise). Reproduced with permission from Lambertsen et al. [88].

Figures 4(a) and 4(b) show how large the changes in arterial blood gases must be for arterial blood gases to provide the key metabolic rate signal driving breathing at maximum exercise (breathing > 100 L·min^−1^). But at maximum exercise (even in endurance trained athletes [31]), PaO_2_ decreases neither transiently nor consistently enough to reach even the FiO_2_ “threshold” in Figure 4(a) that stimulates breathing.

Similarly, PaCO_2_  fails to rise enough. Figures 1 and 2 show that there is barely any reproducible or sustained rise in PaCO_2_  during moderate exercise, and PaCO_2_ actually falls during severe exercise. So there is no PaCO_2_ rise during exercise sufficient to stimulate breathing even to the 20 L·min^−1^ of Figure 4(b). So far, the only observed blood gas changes suitable to provide a metabolic rate signal are in systemic venous blood (Figure 1). Thus, at present there is no obvious arterial factor that rises with metabolic rate and is known to stimulate carotid chemoreceptors in Man.

## 9. Absence of Suitable PaCO_**2**_ Oscillations in Man

Another possibility is that to detect and signal metabolic rate, carotid chemoreceptors might detect some other property of O_2_ or CO_2_, such as the variability in PaO_2_ or PaCO_2_, rather than their absolute levels. Alveolar PCO_2_ in Man does oscillate systematically within the respiratory cycle at rest and is mirrored as oscillations in arterial blood pH and PaCO_2_ [32–34]. It was therefore thought that carotid chemoreceptors might estimate metabolic rate from the rate of rise, or some other mathematical function of the PaCO_2_ oscillation. But there are several difficulties [18] with the CO_2_ oscillation hypothesis. Thus:

- It has not been established whether such oscillations cause the breathing pattern, or whether the breathing pattern causes such oscillations.
- Such oscillations apparently disappear when breathing frequency rises during exercise. Band et al. [32] did find larger pH oscillations at exercise than at rest, but only in one subject and only in the first minute of exercise as tidal volume (but not breathing frequency) increased. Whereas Murphy et al. [33] found that such oscillations disappeared in 4 subjects during the first minute of exercise (0.06 kW) when breathing at 20 breaths per minute (suggesting that it is slow breathing that causes these oscillations and not *vice versa*).
- The oscillation hypothesis is not supported by the demonstration [35] that brief hypoxic pulses (N_2_ in 25–33% of the tidal volume) to the inspirate in 4 subjects, that cause detectable arterial desaturation at the carotid region (earlobe), failed to alter the first relevant respiratory cycle, either at rest or in exercise (≥125 W_ew_), and even if hypoxia (PAO_2_ of 60 mmHg) was used to increase chemoreceptor activity.
- Denervation of carotid chemoreceptors (see [29, 30]—Section 13) apparently fails to prevent breathing matching metabolic rate in Man, yet these are the only known chemoreceptors fast enough to detect such oscillations in Man.

There is no good reason to doubt any of these data. A further difficulty is that it is not yet possible to test whether blood gas changes precede a change in each breath, because sufficiently fast responding arterial electrodes are not generally available [33]. Using breath-by-breath measurement of end tidal PetCO_2_ or PetO_2_ to estimate their corresponding arterial levels preceding each breath is problematic, because end tidal levels are not always reliable estimates of arterial levels at rest and become progressively less so as systemic PvCO_2_ increases with metabolic rate (see Figure 1).

## 10. Absence of Suitable Changes in CO_**2**_ Sensitivity in Man

Another possibility is that whatever initiates exercise might instantly change the sensitivity of arterial chemoreceptors to blood gases [36]. There are two difficulties however with the CO_2_ sensitivity hypothesis. 

 First, since PaCO_2_ fails to rise consistently by more than ~2 mmHg in exercise, the CO_2_ sensitivity of breathing would have to reach at least 50 L·min^−1^·mmHg^−1^ PaCO_2_ to explain breathing reaching 100 L·min^−1^. Yet numerous studies (e.g., [37–41]) show that the measured CO_2_ sensitivity to artificially induced CO_2_ rises in exercise either falls, does not change, or rises to no more than ~7 L·min^−1^·mmHg^−1^ PaCO_2_. Secondly, when faced with the arterial hypocapnia of severe exercise that apparently lowers PaCO_2_ even to below the apnea threshold [42], any important mechanism relying solely on increased CO_2_ sensitivity should cause apnea. Yet clearly apnea does not occur in severe exercise. 

Future experiments could still find some new function of blood gas levels, or of arterial blood (e.g., [H^+^]a—see Section 16), that does change appropriately with metabolic rate and hence could still confirm carotid chemoreceptors as the key metabolic rate sensor driving breathing. But until they do, there remains no variable in arterial blood that rises with metabolic rate and stimulates carotid chemoreceptors.

## 11. Is There an Unknown Substance in Arterial Blood That Rises with Metabolic Rate and Stimulates Carotid Chemoreceptors to Control Breathing in Exercise?

Numerous other blood-borne substances have been considered as candidates to link carotid chemoreceptors to metabolic rate, and the experiments with potassium (K^+^) levels provide a good example of the difficulties with all other suitable candidates meeting the stimulation, ablation, and recording criteria. Thus, while arterial (and venous) plasma K^+^  levels in Man do rise from ~4 to 7 mM between rest and exercise [43], lowering plasma K^+^ levels in Man from 7 to 5 mM fails to alter breathing [44]. Infusing K^+^ intravenously is too dangerous in Man, but infusion to mimic the rise to 5–8 mM in Man produces little increase in breathing in monkeys [45] and none in goats [46]. Furthermore as discussed below, the fact that carotid chemoreceptor denervation fails to prevent breathing matching metabolic rate in Man is evidence that no arterial blood-borne “factor *X*” that stimulates carotid chemoreceptors has been missed. 

## 12. Carotid Chemoreceptor Stimulation Experiments 

The failure to detect a consistent rise in PaCO_2_ or fall in PaO_2_ during exercise has led some to question the relevance to exercise of any experiments that artificially induce hypoxia and hypercapnia to stimulate carotid chemoreceptors. Nevertheless, such experiments are extensively pursued. These show that artificially manipulating the inspired CO_2_ or O_2_ levels to stimulate carotid chemoreceptors fails to cause hyperventilation to the $\dot{\text{V}}\text{e}$ levels anywhere near those seen at maximum exercise (100–150 L·min^−1^). 

Figure 4(a) shows that even lowering FiO_2_ levels to around 4% in resting subjects barely raised minute ventilation >30 L·min^−1^. Yet this is the severest hypoxia tolerable, since humans start to pass out at FiO_2_ levels below 6%. PaO_2_ at maximum exercise does not consistently fall at all, and the lowest levels recorded (75 mmHg [31]) do not even reach the “threshold” to stimulate breathing in Figure 4(a). 

Clearly breathing in Man is more sensitive to small rises in PaCO_2_ than to falls in PaO_2_, for example, artificially lowering eupneic PaCO_2_ by more than ~5 mmHg at rest can cause apnea [42], when untangled from the additional voluntary drive to breathe [47]. Figure 4(b) shows that artificially raising PaCO_2_ to 50 mmHg causes a hyperventilation to only about 20 L·min^−1^. Yet Figures 1 and 2 show that with moderate exercise PaCO_2_ fails to rise consistently above ~45 mmHg, and it actually falls at maximum exercise.

Strictly, the breathing response to hypoxia (Figure 4(a)) is reduced by hypocapnia [48] and accentuated by hypercapnia, that to hypercapnia (Figure 4(b)) is accentuated by hypoxia, [49] and the combination of artificially induced hypoxia and hypercapnia (i.e., asphyxia) is a much more potent stimulant of breathing than either alone. Nevertheless, the failure to detect asphyxic blood gas changes during normal exercise questions the value of attempting to find out what is the maximum minute ventilation achievable with asphyxia. For the same reason, further investigation of Figure 4(a) outside the isocapnic range and of Figure 4(b) outside the iso-oxic range does not appear relevant. Thus, not only has no suitable factor that stimulates carotid chemoreceptors been found to rise in arterial blood, but also artificially inducing large changes in arterial oxygen or carbon dioxide levels in Man to deliberately provide intense stimulation of carotid chemoreceptors fails to stimulate breathing to levels anywhere near those found at maximum exercise.

## 13. Carotid Chemoreceptor Ablation Experiments

Carotid bodies exist in Man [50]. There is not a doubt that hypoxia and hypercapnia stimulate them and rapidly stimulate breathing (Figures 4(a) and 4(b)). In Man after surgical denervation of carotid chemoreceptors bilaterally [51–55], both the rapid component of the hyperventilation to raised PaCO_2_ and the hyperventilation following hypoxia appear absent (although how such hypoxia relates to the FiO_2_ levels in Figure 4(a) is not always clear). Moreover, the hyperventilation to an FiO_2_ of 8% is temporarily abolished by injection of local anaesthetic to block their afferent nerves [56]. Unlike in other species, this abolition of the ventilatory response is permanent in Man [53, 54], although intriguingly such patients retain however a small and residual response to the Dejours test [53, 57] which therefore “*is not wholly attributable to suppression of carotid bodies*” [57]. The initial, rapid time course of the increased breathing to raised PaCO_2_ fits a single exponential [51, 52] so is assumed to involve only one sensor mechanism: the carotid chemoreceptors. 

Available evidence indicates that carotid chemoreceptor ablation in Man does not prevent breathing matching metabolic rate either at rest or during moderate exercise. If carotid chemoreceptors did cause matching, the simplest consequence of their ablation should be marked instability of both blood gases and breathing on a breath-by-breath basis, both at rest and during exercise. Blood gas levels should change so much that other responses should be triggered, for example, unconsciousness at PaO_2_ below ~25 mmHg and/or PaCO_2_ above ~90 mmHg and hypocapnic tetany at PaCO_2_ levels below 20 mmHg (acute hyperoxia being innocuous). Breathing both at rest and during exercise should also be highly unstable and oscillate between periods of apnea and severe hyperventilation. Yet after such ablation in Man, no consistent change has been observed in either arterial blood gases or breathing, neither at rest nor during exercise.

### 13.1. Effects on Breathing of Carotid Chemoreceptor Ablation at Rest

Of the most recent studies, Fatemian et al. [52] found that resting PetCO_2_ levels (45 ± 1 mmHg) in patients with bilateral carotid body removal for glomus tumours (chemodectoma) were 5 ± 1 mmHg higher than in healthy volunteers, whereas other studies with resting patients after bilateral carotid body removal (to treat asthma) found no change in PaCO_2_ or PetCO_2_ versus healthy controls [30, 53, 54], despite asthma itself, if severe enough, undoubtedly causing CO_2_ retention, that is, PaCO_2_ levels at up to 6 mmHg higher than in controls [58, 59]. Dahan et al. [51] found that resting PetCO_2_ levels in 3 chemodectoma patients were 7 mmHg higher (at 41 ± 1 mmHg) after than before denervation. Even if Dahan's study is accepted as the best evidence that PaCO_2_ does rise slightly, their PetCO_2_ of 41 mmHg is still within the normal range in healthy subjects [60].

 It is not therefore obvious that any substantial mismatch between breathing and metabolic rate exists in such patients at rest, nor is their breathing pattern at rest obviously different, although new and more detailed studies of breath-by-breath variability at rest might still reveal subtle deficits.

### 13.2. Effects on Breathing of Carotid Chemoreceptor Ablation during Exercise

**Figure 5:**
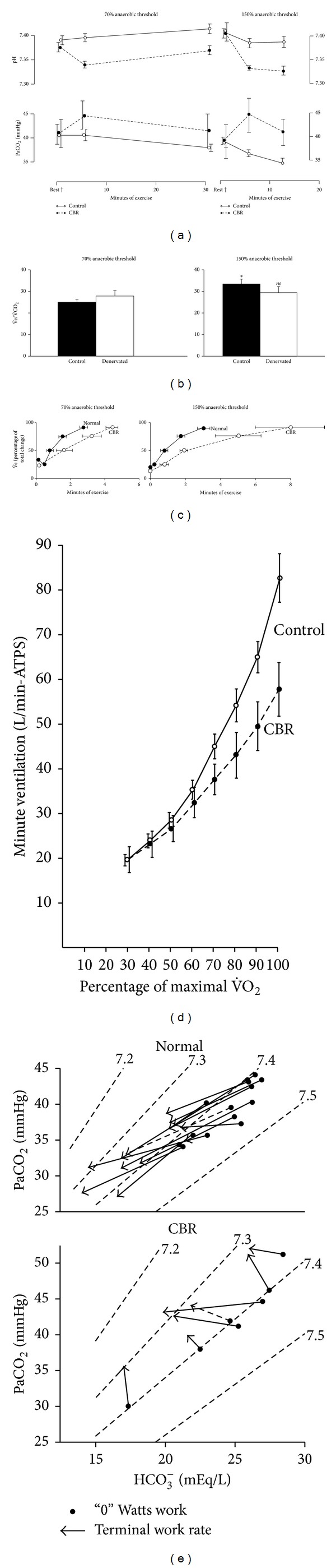
Breathing matches metabolic rate, but with subtle deficits, after carotid chemoreceptor denervation in Man. 5–11 controls at 70% (53 ± 7 W_ew_) or 150% (109 ± 9 W_ew_) anaerobic (lactic acidosis) threshold and 5-6 carotid denervated subjects at 70% (52 ± 7 W_ew_) or 150% (98 ± 17 W_ew_) anaerobic threshold during constant load bicycle ergometry. (a) Mean ± se pH and PaCO_2_ in controls (open circle with filled line) and denervated (filled circle with dashed line). Mean resting PaCO_2_ in controls was 38 ± 2 mmHg and in denervated was 39 ± 3 mmHg. (b) Mean ± se minute ventilation per metabolic rate ($\dot{\text{V}}\text{e}$/$\dot{\text{V}}{\text{O}}_{2}$). *ns P* > 0.05, *P < 0.05  *versus* 70% anaerobic threshold value for that condition. (c) Normalized minute ventilation as a percentage of total change in controls (open circle with filled line) and denervated (filled circle with dashed line) plotted against mean ± se minutes of exercise. (d) Average minute ventilation for percent of maximal $\dot{\text{V}}{\text{O}}_{2}$ for 1 min incremental cycle ergometer work test for 11 control and 6 denervated subjects. Vertical bars at each point are ± 1 se m. (e) PaCO_2_, bicarbonate (HCO_3_^−^), and pH changes in normal (top) and denervated (bottom) subjects at 0 Watts (filled circles) and at terminal (→) work rates for studies described above. Dashed arrows are averages for each group. Diagonal dashed lines are pH isopleths for HCO_3_^−^-pCO_2_ buffer system. Reproduced with permission from Wasserman et al. [30].

No study has established that breath-by-breath control of PaCO_2_ nor the hyperpnea of exercise is obviously abolished after carotid chemodenervation. So far only two groups have tested such patients in exercise. Honda et al. [29] found that subjects could still exercise to 50 W_ew_, but PCO_2_ rose by 3 mmHg (to 46 ± 2 mmHg), whereas Wasserman et al. [30] found (Figure 5) that bilateral denervation during more intense exercise did not prevent breathing from matching metabolic rate, that is,

* “the carbon dioxide … blood gas tensions … remained unchanged … in exercise” at 50 *W*_*ew*_  (*n* = 7). *

and

*“a marked transient overshoot in the PaCO_2_ … prior to attaining a phase III response in which PaCO_2_ was indistinguishable from resting levels” *[11]* at 98 *W*_ew_ exercise (*n* = 6)* [30]*. *

Note *precisely* what the PaCO_2_ levels are in Figure 5(a) and compare them with Figures 1, 2(b), and 2(c). 

This negative result appears validated by their positive result [54] that the stimulation of breathing with an FiO_2_ of 12% was absent in their carotid body denervated patients. In fact, Figure 4(a) indicates that using an FiO_2_ of ~8% [56] would be definitive, but the precise FiO_2_ “threshold” at which the stimulation of breathing first occurs appears never to have been deliberately measured with modern measurement and statistical analysis techniques. Thus, ablation appears technically successful, and no obvious measurement errors occurred. 

It is not reasonable to propose that such ablation makes the control system too abnormal to be relevant, because it is not particularly abnormal. Moreover, although such ablation apparently abolishes the stimulation of breathing by hypoxia, its ablation is no hindrance, since such hypoxia does not occur during exercise.

Correct interpretation of these experiments is crucial. The usual interpretation is that “*carotid chemoreceptors must still be important and redundancy must exist.*” But a more realistic interpretation is “*inconclusive without further experiments,*” because these results demonstrate that either the chemoreceptors must be one of at least two important mechanisms that sense metabolic rate (i.e., are duplicating) or have no importance. 

In either case, this shows that at least one mechanism other than carotid chemoreceptors must also account for breathing matching metabolic rate. This other mechanism remains unidentified, and, until it is, we cannot pursue experiments to distinguish its role from that of carotid chemoreceptors and hence to distinguish between carotid chemoreceptors being redundant or irrelevant.

## 14. Aortic and Central Chemoreceptors Too Fail to Sustain Breathing after Ablation of Carotid Chemoreceptors in Man

How do carotid chemodenervated patients maintain relatively normal breathing at rest during sleep, wakefulness and exercise? This question is still unanswered, but it appears unlikely that the second mechanism involves either aortic or central chemoreceptors acting as the key metabolic rate sensor, because they too have no known means of measuring metabolic rate.

Aortic bodies do exist in humans [50]; they may have cardiovascular effects [61] but have no known effects on breathing in Man. Therefore, there is no evidence that they can act as the metabolic sensor. Thus, carotid chemodenervation (while the aortic chemoreceptors are still intact) apparently abolishes the breathing response to hypoxia permanently [51, 52, 54, 55], and that additional bilateral anaesthesia of the vagus nerve (in which aortic afferents travel) produces no further deficits [56]. So not only do aortic chemoreceptors not affect breathing in Man, but also they too have no known arterial blood factor to stimulate them as metabolic rate increases during exercise.

Following carotid chemodenervation, the residual slow ventilatory response to hypercapnia [51, 52, 62] is attributed to the existence in Man of central chemoreceptors (rather than the alternative explanation that only hypercapnia stimulates aortic chemoreceptors in Man and that they then stimulate breathing only after an unusually long latency). Available evidence indicates that central chemoreceptors have no means of sensing metabolic rate and they too are in the wrong place to detect metabolically, produced CO_2_ (Figure 3). Thus they are not stimulated by hypoxia, and the blood brain barrier supposedly prevents arterial blood acidity from directly stimulating them and introduces a too long time delay for them to control breathing usefully. Finally, the hyperventilation of severe exercise should make the CSF both hypocapnic and alkalotic (as it does in other species) and hence should further unload central chemoreceptors and induce apnea if their role was important. Thus, any role for central chemoreceptors during more intense exercise appears to be eliminated.

## 15. Do Carotid Chemoreceptors Mediate Just the Hyperventilation of Exercise in Man?

**Figure 6:**
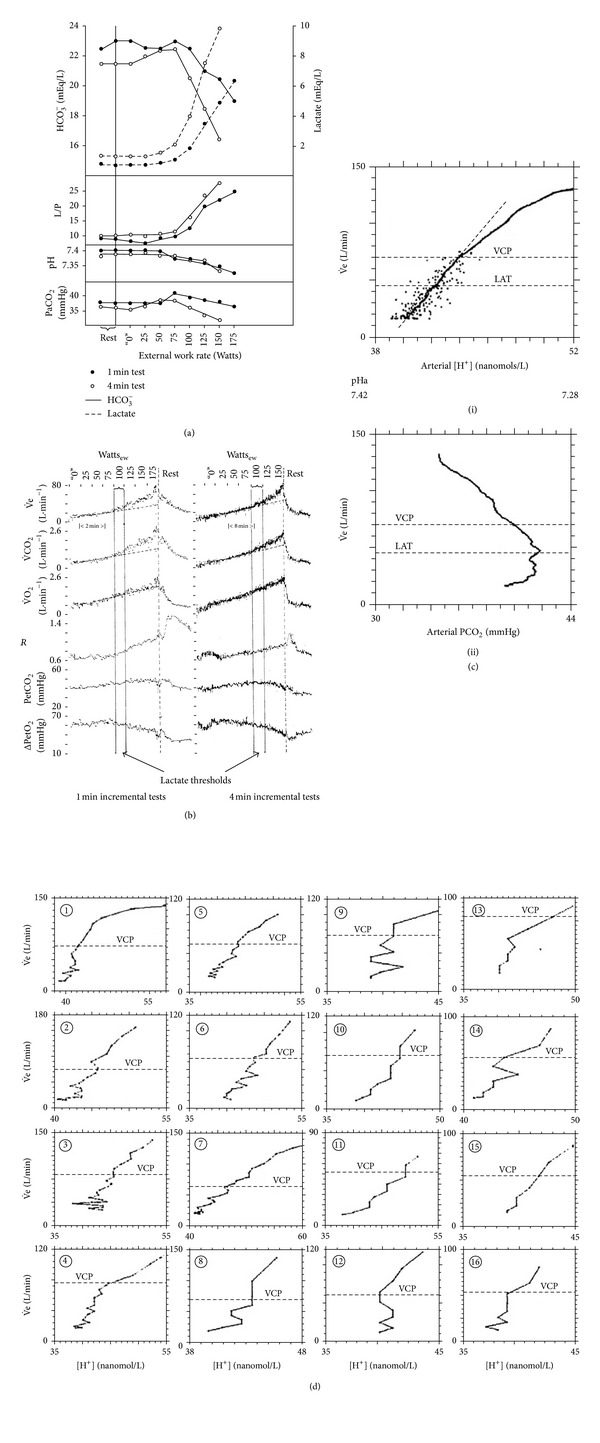
(a) The coincidence of the lactate threshold and hyperventilation (falling PaCO_2_) in normal subjects. Mean lactate (- - -), bicarbonate (—), lactate/pyruvate ratio, pHa, and PaCO_2_ levels during 1 min (*∙*) and 4 min (∘) incremental work tests (*n* = 61) at external work rates (W_ew_) indicated. (b) The coincidence of the lactate threshold and hyperventilation in normal subjects. Mean ($\dot{\text{V}}\text{e}$), CO_2_ production ($\dot{\text{V}}\text{C}{\text{O}}_{2}$), metabolic rate ($\dot{\text{V}}{\text{O}}_{2}$), gas exchange ratio (*R*), PetCO_2_, and change in PetO_2_ at external (W_ew_) work rates indicated in the same subjects as (a). (a) and (b) reproduced with permission from Wasserman et al. [89]. (c) The correlation between breathing ($\dot{\text{V}}\text{e}$) and [H^+^]a or PaCO_2_ during exercise averaged over 16 subjects. $\dot{\text{V}}\text{e}$ is scaled for each study to a VCP at 70 L/min (the mean $\dot{\text{V}}\text{e}$ at the VCP for all subjects). The lowest point for each study is scaled to 17 L/min (the mean unloaded pedalling $\dot{\text{V}}\text{e}$ for all subjects). The diagonal dashed line is the regression line of the averaged data in the below VCP region. The labelled horizontal dashed lines indicate the mean $\dot{\text{V}}\text{e}$ values at the VCP (the point when $\dot{\text{V}}\text{e}$ begins to accelerate out of proportion to the increase in $\dot{\text{V}}\text{C}{\text{O}}_{2}$ and the LAT (lactic acidosis threshold)). The other labelled horizontal dashed line indicates the mean $\dot{\text{V}}\text{e}$ at the LAT for the 16 subjects. The average $\dot{\text{V}}\text{e}$ is linearly related to [H^+^] up to the VCP, as indicated by *r* > 0.99. The individual points are the transformed data points used in calculating the average curve up to the VCP. The data for all 16 subjects are used in this calculation. Above the VCP, subjects drop out because of differences in exercise tolerance. The remaining studies are shifted along the [H^+^] axis to provide an accurate representation of the remaining data. The number of subjects determining the average curve decreases to 15, 15, 8, 5, and 4 at [H^+^] values of 45, 46, 48, 49, and 50 nmol/L, respectively. (d) Zigzags in the correlation between [H^+^]a and breathing $\dot{\text{V}}\text{e}$ during exercise in 16 individual subjects. The points on the respective curves identify the blood sample points. Each plot starts at the beginning of linearly increasing work rate and ends at the cessation of exercise after the highest [H^+^]a and $\dot{\text{V}}\text{e}$ are reached. The $\dot{\text{V}}\text{e}$ at the ventilatory compensation point (VCP), defined as the disproportionate increase in $\dot{\text{V}}\text{e}$ relative to $\dot{\text{V}}\text{C}{\text{O}}_{2}$ as work rate increases (start of hyperventilation with respect to CO_2_), statistically determined, is indicated by the horizontal line. (c) and (d) reprinted from [74] with permission from Elsevier.

Although Wasserman et al.'s [30] chemodenervated asthmatics retained their hyperpnea during exercise, one positive consequence of ablation was their inability to change breathing at the normal speed (Figure 5(c)) and supposed inability to hyperventilate during the metabolic lactoacidosis. Whether this is a partial or a complete inability to hyperventilate may be judged by detailed examination of Figures 5(d) and 5(e). This is often proposed as evidence that the carotid chemoreceptors normally cause the hyperventilation of exercise, that is, a respiratory compensation for metabolic acidosis [11, 30], but not the hyperpnea itself. Although there is no direct evidence to refute this hypothesis, there remain several outstanding difficulties. Thus:

- The coincidence (Figures 6(a) and 6(b)) in healthy subjects of the simultaneous occurrence at the same exercise intensity of the “lactate threshold” and hyperventilation may just reflect a correlation rather than a causal relationship [36] because it is not always found, nor does it always persist after training [63], and there is no correlation between the severity of the acidosis and the size of the hyperventilation [64].
- The zigzag in the relationship between [H^+^]a and $\dot{\text{V}}\text{e}$ in individuals, occurring in 13/16 subjects (Figure 6(d)), is inconvenient.
- Patients who cannot produce lactic acid (McArdle's syndrome) appear to have a similar hyperventilation (i.e., have a nonlinear increase in breathing) at about the same relative exercise intensity [65]. Nevertheless, since $\dot{\text{V}}{\text{O}}_{2}$ max. in such patients is, however, so much lower (25–46% of those in healthy controls), the validity of this comparison may be questioned.
- Experimentally induced changes in acid production do not always produce appropriate changes in the hyperventilation [18, 66].
- This proposal predicts that the hyperventilation should be abolished during exercise by unloading the carotid chemoreceptors with sufficient hyperoxia, but this has not yet been demonstrated [67].
- The metabolic acidosis of exercise is never fully compensated by the hyperventilation [67, 68]. It is not obvious why carotid chemoreceptors should fail to achieve complete respiratory compensation, whereas this failure is irrelevant if the primary purpose of the hyperventilation has nothing to do with respiratory compensation.

Furthermore, there remain several alternative explanations for the results in Figure 5. Thus

- This positive effect of ablation—the failure to hyperventilate—might have been some property of these particular asthmatics. It was never established by how much these particular asthmatics could hyperventilate before their chemodenervation (and there appear to be no definitive studies showing that asthma alone cannot also cause this), although such experiments are still possible as this operation is still performed [51].
- The failure to hyperventilate might be because brain temperature failed to rise as much in the chemodenervated asthmatics. To allow for their lower “lactate threshold,” they were exercised at a 10% lower intensity (98 ± 17 W_ew_) than the intact controls (109 ± 9 W_ew_) [30]. It is possible that it is hyperthermia instead that causes the hyperventilation to increase evaporative heat loss [69]. Any additional ventilatory loss of metabolic acid may be merely fortuitous (and this would explain why the hyperventilation never fully compensates for the metabolic acidosis). Exercise (90 W_ew_ or more) can raise CNS (tympanic membrane) temperature by ~1°C [70, 71]. Such hyperthermia induced at rest can itself cause sufficient hyperventilation in Man to lower PaCO_2_ below 25 mmHg [71, 72]. Moreover, preventing temperature rising during exercise at 1 kW prevents PaCO_2_ falling from 40 to 30 mmHg [73].

All these difficulties could easily be dismissed by a new study demonstrating that such patients do hyperventilate before denervation (but not afterwards), and that they have the same CNS temperature rises during exercise as those in intact subjects. But without such a study, it is surprisingly difficult to provide definitive evidence that the carotid chemoreceptors mediate the hyperventilation of exercise.

## 16. Could New Evidence for Acidemia below the Lactate Threshold Provide the Stimulus to Carotid Chemoreceptors That Drives Breathing in Exercise?

It has always been believed that metabolic acidosis itself causes the hyperventilation, even if not the hyperpnea of exercise. Thus, Figure 6(a) shows that acidemia did not occur in low intensity exercise, that is, below the lactate threshold when only hyperpnea occurred. Here the rate of arterial blood gas sampling was however unspecified. Moreover after exercise, the decline in breathing did not apparently correlate with the changes in arterial blood acidity (e.g., [68]). But the latest work of Wasserman et al. [74], involving rapid arterial sampling during exercise (6 per minute) and improved blood gas measurement technology and expression of acidity in [H^+^]a units rather than the traditional but less sensitive pH notation, now challenges this belief. It shows (see Figure 6(c)) that there may after all be a rise in [H^+^]a in exercise below the lactate threshold. This may also be indicated in their earlier data (see Figure 2(b) and [75]). This relationship however is still only an intriguing correlation and does not establish any causal relationship between [H^+^]a, carotid chemoreceptors, and breathing during rest or exercise. Could [H^+^]a be the missing link between metabolic rate and carotid chemoreceptors? To establish a causal relationship, new stimulation and ablation experiments are still required to test whether:

- infusing H^+^ stimulates breathing at rest (analogous to the “infusions” of O_2_ & CO_2_ of Figures 4(a) and 4(b)),
- infusion at rest to achieve the [H^+^]a levels in Figure 6(c) will mimic the breathing levels in Figure 6(c),
- preventing the [H^+^]a rise during exercise prevents the increase in breathing.

The main problem for this hypothesis too however is still the fact that since carotid body denervation fails to prevent breathing matching metabolic rate during exercise, how can [H^+^]a, acting on carotid chemoreceptors alone, provide the link between metabolic rate and breathing during exercise?

## 17. Possible Outcomes with Future Recording Experiments in Man

All the above difficulties could be resolved if a new technique is found to record neural activity directly from peripheral arterial chemoreceptors in Man. Intriguingly all the above evidence could fit either of the two possible outcomes from such a recording experiment.

(A) Recording might indicate that their neural activity during maximum exercise is barely different from that at rest. In this case, combining these results with the available evidence from the other 2 key conditions (the absence of any arterial stimulus to carotid chemoreceptors and the inability of carotid chemoreceptor ablation to prevent matching) would indicate that carotid chemoreceptors are not the key metabolic rate sensor. But even these negatives would be finally validated only when the mechanism that does act like a metabolic rate sensor is discovered.

(B) Recording might indicate instead that their neural activity during exercise does increase in proportion to metabolic rate. Combining the results of the available evidence from the 3 key experimental conditions described above would confirm the opposite conclusion that carotid chemoreceptors are the key metabolic rate sensor. In this case, the absence of a carotid arterial stimulus would be an example of “*absence of evidence is not evidence of absence.*” It would suggest that a carotid arterial stimulus must exist and will be found when new research adopts different approaches.

 The failure of carotid chemoreceptor denervation to prevent matching now favours duplication, that is they and at least one other mechanism both act as key metabolic rate sensors. It would then be necessary to test whether matching is prevented by ablation only of the second mechanism (carotid chemoreceptors therefore being irrelevant) or only by ablation of both (carotid chemoreceptors therefore being important but duplicating).

The correlation between their neural activity and metabolic rate implies causation, which could then be established from the appropriate outcome of the other 2 conditions.

## 18. Do Carotid Chemoreceptors Act Only to Fine-Tune Breathing during Exercise in Man? 

Although at least one factor other than carotid chemoreceptors must act equally well as carotid chemoreceptors as a metabolic rate sensor, carotid chemoreceptors still could be and are often proposed [76] to operate as mismatch sensors to fine-tune every breath on a breath-by-breath basis. While they have the potential to do so, there is no evidence yet that they do act in this way.

Carotid chemoreceptors *potentially* have this ability because, as already discussed, artificially inducing hypoxia or hypercapnia always stimulates breathing. Moreover carotid chemoreceptor discharge in anaesthetized or unanaesthetised animals changes fast enough to detect changes in PaCO_2_ between breaths [77], and artificially applying large changes in PaO_2_ does affect breathing during exercise. Thus:

- Hypoxia (FiO_2_ ~12%) accelerates the speed of the breathing response [78].
- Hyperoxia ([79] the “Dejours test”) causes a small reduction and slowing of the kinetics of breathing [78, 80] (although it has never been established how equivalent is the Dejours test to a proper carotid chemoreceptor denervation).
- Carotid denervated patients may have a slower breathing response to exercise (see Figure 5(c)), have reduced sensitivity to artificially raising CO_2_, and fail to respond to the Dejours test [14, 81].

Such evidence has always sustained the belief that somehow carotid chemoreceptors “must” be important in exercise [76]. And the mismatch hypothesis is particularly attractive, since the required blood gas changes during exercise need only involve PaCO_2_ and could only be of a few mmHg. Furthermore, mismatch need not occur consistently, because whatever sensed metabolic rate might be so successful that mismatch rarely occurs during exercise, and therefore, carotid chemoreceptors normally have little to do.

The key question however is whether they *do* act as mismatch sensors that make any important contribution to breath-by-breath control of breathing during exercise. Establishing this requires new experiments measuring the incidence of breaths mismatching metabolic rate during exercise, demonstrating that each mismatched breath is followed by a PaCO_2_ change, and demonstrating that this PaCO_2_ change causes a subsequent augmented breath (or two) to restore PaCO_2_. At present the available evidence does not support the hypothesis that they do act to fine-tune every breath during exercise. Thus:

- Carotid denervation experiments failed to reveal any obvious changes in breathing pattern or blood gases during exercise [29, 30]; that is, removal of the receptors necessary to mediate fine-tuning appears not to disturb greatly the control of breathing during exercise.
- Strange-Petersen et al.'s [35] stimulation experiments in 4 subjects using brief pulses of hypoxia failed to alter the next breath (see Section 9).
- The best recording experiment available so far to test this hypothesis, by making frequent and subtle blood gas measurements (at least breath-by-breath) using intra-arterial pH electrodes [33]—see Section 9—reveals no evidence of a signal which carotid chemoreceptors could use for fine-tuning.

## 19. Are Carotid Chemoreceptors Important in Learning Postnatally How to Control Breathing?

There is one last and ingenious hypothesis [82, 83]. Since breathing does match metabolic rate so well in adults, is matching present at birth, or only learnt postnatally? If learnt, there has to be an early postnatal period for learning, when breathing fails to match metabolic rate well. Episodes of pronounced mismatch should therefore be more readily detectable in neonates. Such mismatch would result in proportionate blood gas error signals [82, 83], and the carotid chemoreceptors would be the essential mismatch sensors to detect them and to stimulate breathing accordingly. The brain could then learn to become a metabolic rate sensor by anticipating the breathing required for every possible physical exertion in order to minimize these mismatch error signals. Such learning may become so successful that by adulthood mismatch almost never occurs. Hence, the carotid chemoreceptors would not normally be stimulated in exercise, while always being available to signal if mismatch ever occurred. Obviously no experiments have yet been devised to test this hypothesis in Man, and interpretation of such tests in neonates of other species is complicated by the fact that in other species carotid chemoreceptors may not be the only peripheral chemoreceptors that stimulate breathing.

## 20. Difficulties in Interpreting Recent Animal Experiments Studying Carotid Chemoreceptors 

Ultimately it is not acceptable to combine key arguments based on experiments across species, for example, a stimulation experiment from a dog, a recording experiment from a goat, and an ablation experiment from a cat. Unfortunately this often has to be done [15, 16] because only information across species is available. Ultimately the case for whatever acts as the key metabolic rate sensor must be established by stimulation, ablation, and recording in each species separately, as this review considers for Man. There are difficulties for all other mammalian species (for reviews see Section 1) in establishing definitively if carotid chemoreceptors are the key metabolic rate sensors that mediate matching. Recent and ingenious experiments on dogs show some of the equal difficulties in establishing a role for carotid chemoreceptors in other species. 

Blain et al. [84] performed a series of experiments on 4 unanaesthetised and resting dogs with carotid bodies on one side denervated and the other side being experimentally unloaded by extra corporeal perfusion with hyperoxic (PaO_2_ > 500 mmHg) and hypocapnic (PaCO_2_ ~20 mmHg) blood. Unloading transiently decreased ventilation but, while unloading persisted, ventilation partially recovered (after 50–70 s). Furthermore, in 8 resting dogs such unloading decreased the slope (by 19%) of the breathing response to artificially lowering PetCO_2_ (by 10–12 mmHg below its eupneic level, [85]), whereas stimulation at rest with artificially hypoxic arterial blood (PaO_2_ of 40 mmHg) increased the slope by 223%. 

Detecting any effects on breathing with such manipulations is all the more remarkable because aortic chemoreceptors also stimulate breathing in dogs and enhance the breathing response to carotid body stimulation [86]. Thus, information from intact aortic chemoreceptors will contradict that from such manipulated carotid chemoreceptors.

It is still unclear precisely what these ingenious experiments reveal about the role of carotid chemoreceptors as metabolic rate or mismatch sensors in resting dogs. If it were already established definitively that carotid chemoreceptors are the key metabolic rate sensors in dogs, these experiments provide elegant confirmation. But this is still unclear in dogs. Without an arterial blood gas (or other) change to link carotid chemoreceptors with metabolic rate in dogs, these results are equally consistent with carotid chemoreceptors not being the key metabolic rate sensors. Instead they also confirm merely that breathing in dogs is alterable by withdrawing or adding another stimulus and provide no evidence about their role in breathing matching metabolic rate during exercise.

## 21. Conclusions

The only compelling evidence that carotid chemoreceptors are important in controlling breathing during exercise in Man is the fact that they are the only peripheral chemoreceptors ever found in Man that are stimulated by hypoxia, which in turn stimulates breathing. Since neither arterial hypoxia nor hypercapnia occurs during exercise to stimulate carotid chemoreceptors in Man and bilateral carotid chemoreceptor denervation in Man, fails to prevent breathing matching metabolic rate, we still can neither confirm nor refute the belief that carotid chemoreceptors are important in controlling breathing during exercise in Man. 

There is still huge controversy over whether carotid chemoreceptors have any importance in controlling breathing during exercise, despite ~80 years of research. Remarkably two reviews in the same journal in 2012 [15, 16] still draw very different conclusions about their role. This review identifies what evidence is necessary to establish how important are carotid chemoreceptors in controlling breathing in exercise in Man, the species in which we have most interest. Without being able to record from carotid chemoreceptors during exercise, the best conclusion so far is that we still cannot distinguish between carotid chemoreceptors being one of two important but duplicated mechanisms, or having no importance in controlling breathing during exercise in Man.
